# Pancreatic Fibrosis and Chronic Pancreatitis: Mini-Review of Non-Histologic Diagnosis for Clinical Applications

**DOI:** 10.3390/diagnostics10020087

**Published:** 2020-02-07

**Authors:** Chung-Tsui Huang, Cheng-Kuan Lin, Tzong-Hsi Lee, Yao-Jen Liang

**Affiliations:** 1 Division Gastroenterology, Department of Internal Medicine, Far-Eastern Memorial Hospital, No.21, Sec. 2, Nanya S. Rd., Banciao Dist., New Taipei City 220, Taiwan; 0989206131ng@gmail.com (C.-T.H.); widelin@mail.femh.org.tw (C.-K.L.); thlee@mail.femh.org.tw (T.-H.L.); 2 Graduate Institute of Applied Science and Engineering, College of Science and Engineering, No.510, Zhongzheng Rd., Xinzhuang Dist., New Taipei City 24205, Taiwan

**Keywords:** pancreatic fibrosis, early chronic pancreatitis, non-histologic diagnosis

## Abstract

Pancreatic fibrosis is the dominant reversible pathological change and diagnostic factor in early chronic pancreatitis, defined by a mechanistic approach proposed in 2016. Main guidelines for chronic pancreatitis were published by the American Pancreas Association in 2014, the Japanese Society of Gastroenterology in 2015, and United European Gastroenterology in 2017. All three sets of guidelines mentioned that the staging of chronic pancreatitis is important but challenging. There are various image modalities for the non-histologic diagnosis of pancreatic fibrosis: (1) shear wave elastography, such as an acoustic radiation force impulse with a cut-off value of 1.4 m/s; (2) strain elastography using grades of strain; (3) endoscopic ultrasonography using the Rosemont criteria or endoscopic ultrasound criteria for early chronic pancreatitis proposed by the Japan Pancreas Society; (4) computed tomography using the Hounsfield scale or number of micro-calcifications; and (5) magnetic resonance imaging using the apparent diffusion coefficient and the T1w flash and T2w HASTE sequences. The clinical applications are to (1) evaluate pancreatic tumors and inflammatory disease; (2) monitor dyspepsia with early chronic pancreatitis; (3) monitor individuals with a high risk of pancreatic cancer; (4) analyze a fatty pancreas with fibrosis; (5) predict a fistula after pancreatic surgery; and (6) predict outcomes for chronic pancreatitis or pancreatic cancer. The selection of tools will be dependent on the clinical scenario. Conclusion: There are various modalities for the non-histologic diagnosis of pancreatic fibrosis. The selection of the optimal device will be dependent on the clinical scenario.

Chronic pancreatitis is a major disease in the digestive system with severe complications in the end stage, including exocrine and endocrine insufficiency and pancreatic duct adenocarcinoma. An epidemiological study showed that a prevalence of chronic pancreatitis is between 36.9 and 41.8 per 100,000 people in America [1] and Japan [2] and up to 120 per 100,000 people in Europe [3]. Recent guidelines for chronic pancreatitis can be traced to the American Pancreas Association published in 2014 [4], the Japan Pancreas Society in 2015 [5], and United European Gastroenterology in 2017 [3]. These guidelines mentioned that the staging of chronic pancreatitis is important but challenging. Therefore, an international consensus statement focusing on early chronic pancreatitis was published in 2018 [6]. This consensus reappraised a mechanistic definition proposed in 2016 for chronic pancreatitis [7]. The mechanistic definition proposed a concept of early chronic pancreatitis, which can exhibit the reversible structural fibrotic change of late stage chronic pancreatitis. The fibrosis of pancreatic parenchyma can be an early stage surrogate for chronic pancreatitis. With this in mind, endoscopic ultrasound (EUS) coupled with elastography would currently be the most sensitive diagnostic modality for detecting pancreatic parenchymal fibrosis.

Pancreatic fibrosis and atrophy are two major pathological changes of chronic pancreatitis [4]. Chronic pancreatitis is a risk factor of pancreatic duct adenocarcinoma, which shows a trend of increasing incidence [8,9]. The basic study of pancreatic fibrosis has advanced toward elucidating the pathogenesis and finding a possible therapeutic agent [10,11,12]. The stellate cell has been found to regulate the processes of pancreatic fibrosis. In addition, pancreatic fibrosis is a surveillance surrogate for individuals with a high risk of pancreatic duct adenocarcinoma [13].

There are histologic and non-histologic approaches for the diagnosis of pancreatic fibrosis. Image localization of pancreatic tissue fibrosis is valuable before biopsy, because the fibrotic change is probably focal. Pancreatic parenchyma tissue can be obtained by EUS-guided biopsy, percutaneous biopsy, or surgical biopsy. The non-histologic diagnosis of pancreatic fibrosis is an image-based method using different bio-physical mechanisms, including ultrasound elastography, computed tomography (CT), and magnetic resonance imaging (MRI) [14]. There are two types of ultrasound elastography—strain elastography through endoscopic [15] or transcutaneous routes [16] and shear wave elastography using only the transcutaneous route [17]. CT and MRI are both non-invasive transcutaneous image reconstructions.

The selection of different tools for the non-histologic diagnosis of pancreatic fibrosis is disease specific and scenario oriented. CT is used for predicting the post-operative pancreatic fistula complication by the CT value [18] or micro-calcifications. One study designed the computed tomography as an image prediction tool for severe pancreatic fibrosis and pain relief after surgical pancreatic resection in chronic pancreatitis patients. This study found that a predictor is calcification, especially when the number is > 10 [19].

Shear wave elastography for pancreatic fibrosis can be used in the following conditions: (1) differentiation of pancreatic cystic or solid tumor [20,21,22,23]; (2) acute and chronic pancreatitis [20,24,25,26,27,28]; (3) cystic fibrosis in the pancreas [14,29]; (4) prediction of post-operation pancreatic fistula [30]; and (5) surveillance of pancreatic fibrosis in a fatty pancreas [31]. The cut-off value of pancreatic fibrosis using acoustic radiation force impulse is 1.4 m/s [32]. It is well known that EUS can detect subtle parenchymal morphologic changes of pancreatic fibrosis in the early stage of chronic pancreatitis. It is evident that the EUS criteria of chronic pancreatitis have a histologic association with pancreatic fibrosis [4]. The commonly used EUS criteria for pancreatic fibrosis in chronic pancreatitis are the Rosemont criteria [33,34] and the early chronic pancreatitis EUS criteria defined by the Japan Pancreas Society [35,36]. EUS can be used for the surveillance of dyspeptic patients with early chronic pancreatitis and provides therapeutic guidance [37,38,39]. In these clinical studies, the diagnostic criteria of early chronic pancreatitis proposed by the Japan Pancreas Society have two parts. The first part details the clinical or functional criteria: (1) recurrent upper abdominal pain (two or more attacks); (2) abnormal serum or urine enzyme levels; (3) abnormal exocrine function; and (4) continuous heavy drinking (>80 g/d). The second part details the image features of EUS or endoscopic retrograde cholangiopancreatography (ERCP). The EUS features are (1) lobularity with honeycombing; (2) lobularity without honeycombing; (3) hyperechoic foci without shadowing; (4) Stranding; (5) cysts; (6) dilated side branches; and (7) hyperechoic main pancreatic duct margin. ERCP shows irregular dilatation of more than three duct branches [35]. In these references, the diagnosis required two or more clinical features and two or more EUS features.

Secretin-enhanced magnetic resonance cholangiopancreatography can depict the ductal system of the pancreas but lacks further analysis of parenchyma. A recent study, specified as magnetic resonance imaging as a non-invasive method for the assessment of pancreatic fibrosis (MINIMAP), was designed to incorporate both the parenchymal and ductal features of chronic pancreatitis. The aim of this study was to make a MRI-based quantitative scoring system for pancreatic fibrosis [40]. One recent study showed that body composition and pancreatic fibrosis could both be factors associated with quality of life and treatment outcomes in patients with chronic pancreatitis or pancreatic duct adenocarcinoma. Pancreatic fibrosis can be calculated by MRI images using T1 signals, apparent diffusion coefficient maps, and T1w flash and T2w HASTE sequences [14,41].

Here, we explain the demerits of each of the modalities. Endoscopic strain elastography is an operator-dependent invasive procedure without quantitative results. Shear wave elastography is a point-targeted evaluation and is occasionally difficult to perform in an extremely obese patient. The CT examination exposes the patient to radiation and is unable to detect the early structural change of pancreatic fibrosis. MRI is a high-cost image device and is superior in the pancreatic ductal system but not in detecting the subtle morphologic change of parenchyma in the current technique.

In addition to the image diagnostic methods, serologic research for the diagnosis of chronic pancreatitis with parenchymal fibrosis is evolving. One specific serum circulating immune signature was proved to diagnose chronic pancreatitis, pancreatic duct adenocarcinoma, and recurrent acute pancreatitis with an area under the curve of approximately 0.77~0.86 [42]. For serologic predictors of chronic pancreatitis with fibrosis, the very low level of pancreatic serum enzymes (amylase or lipase) can be regarded as a marker [43,44]. Chromogranin-A is an acidic protein and one member of the granin family of neuroendocrine secretory proteins and is located in the cells of the endocrine and nervous systems. It can be used as a plasma marker or prognostic factor for pancreatic neuroendocrine tumor [45,46] and advanced pancreatic cancer [47,48] and, theoretically, for both diseases with concomitant focal pancreatic fibrosis. Besides, the association between pancreatic fibrosis and the level of chromogranin-A in tissue has had a couple of research findings as follows: (1) an increase in chromogranin A-positive but hormone-negative endocrine cells in the pancreatic tissue of cystic fibrosis patients [49] and chronic pancreatitis patients [50] and (2) an increased expression of chromogranin-A in the duodenal mucosa of pancreatic fibrosis patients [31].

Conclusively, there are various modalities for the non-histologic diagnosis of pancreatic fibrosis. The selection of the optimal device will be dependent on the clinical scenario. The various image modalities for the non-histologic diagnosis of pancreatic fibrosis and clinical applications are summarized in Table 1.

## Figures and Tables

**Table 1:** Various image modalities for the non-histologic diagnosis of pancreatic fibrosis and clinical applications.

Image Modalities	Parameters	Clinical Scenario Application
Trans-abdomen strain elastography	grade of strain	pancreatic tumors
Trans-abdomen shear wave elastography (ARFI)	≥ 1.4 m/s	pancreatic tumors dyspepsia surveillance fatty pancreas
EUS	Rosemont criteria, early CP criteria	pancreatic tumor high-risk population surveillance dyspepsia surveillance
EUS: strain elastography	grade of strain	pancreatic tumors
CT	CT value, micro-calcification	predicting fistula after pancreatic resection operation
MRI	ADC, T1, T2 different sequences	predicting therapeutic outcomes and quality of life for chronic pancreatitis or pancreatic cancer

ARFI—acoustic radiation force impulse, EUS—endoscopic ultra-sonography, CP—chronic pancreatitis, CT—computed tomography, MRI—magnetic resonance image, ADC—apparent diffusion coefficient.

## References

[1] Yadav D., Timmons L., Benson J.T., Dierkhising R.A., Chari S.T. (2011). Incidence, Prevalence, and Survival of Chronic Pancreatitis: A Population-Based Study. Am. J. Gastroenterol..

[2] Lin Y., Tamakoshi A., Matsuno S., Takeda K., Hayakawa T., Kitagawa M., Naruse S., Kawamura T., Wakai K., Aoki R. (2000). Nationwide epidemiological survey of chronic pancreatitis in Japan. J. Gastroenterol..

[3] Lohr J.M., Dominguez-Munoz E., Rosendahl J., Besselink M., Mayerle J., Lerch M.M., Haas S., Akisik F., Kartalis N., Iglesias-Garcia J. (2017). United European Gastroenterology evidence-based guidelines for the diagnosis and therapy of chronic pancreatitis (HaPanEU). United Eur. Gastroenterol. J..

[4] Conwell D.L., Lee L.S., Yadav D., Longnecker D.S., Miller F.H., Mortele K.J., Levy M.J., Kwon R., Lieb J.G., Stevens T. (2014). American Pancreatic Association Practice Guidelines in Chronic Pancreatitis: Evidence-based report on diagnostic guidelines. Pancreas.

[5] Ito T., Ishiguro H., Ohara H., Kamisawa T., Sakagami J., Sata N., Takeyama Y., Hirota M., Miyakawa H., Igarashi H. (2016). Evidence-based clinical practice guidelines for chronic pancreatitis 2015. J. Gastroenterol..

[6] Whitcomb D.C., Shimosegawa T., Chari S.T., Forsmark C.E., Frulloni L., Garg P., Hegyi P., Hirooka Y., Irisawa A., Ishikawa T. (2018). International consensus statements on early chronic Pancreatitis. Recommendations from the working group for the international consensus guidelines for chronic pancreatitis in collaboration with The International Association of Pancreatology, American Pancreatic Association, Japan Pancreas Society, PancreasFest Working Group and European Pancreatic Club. Pancreatology.

[7] Whitcomb D.C., Frulloni L., Garg P., Greer J.B., Schneider A., Yadav D., Shimosegawa T. (2016). Chronic pancreatitis: An international draft consensus proposal for a new mechanistic definition. Pancreatology.

[8] McGuigan A., Kelly P., Turkington R.C., Jones C., Coleman H.G., McCain R.S. (2018). Pancreatic cancer: A review of clinical diagnosis, epidemiology, treatment and outcomes. World J. Gastroenterol..

[9] Matsubayashi H., Ishiwatari H., Sasaki K., Uesaka K., Ono H. (2019). Detecting Early Pancreatic Cancer: Current Problems and Future Prospects. Gut Liver.

[10] Ceyhan G.O., Friess H. (2015). Pancreatic disease in 2014: Pancreatic fibrosis and standard diagnostics. Nat. Rev. Gastroenterol. Hepatol..

[11] Apte M., Wilson J. (2004). Mechanisms of Pancreatic Fibrosis. Dig. Dis..

[12] Shimizu K. (2008). Mechanisms of pancreatic fibrosis and applications to the treatment of chronic pancreatitis. J. Gastroenterol..

[13] Thiruvengadam S.S., Chuang J., Huang R., Girotra M., Park W.G. (2019). Chronic pancreatitis changes in high-risk individuals for pancreatic ductal adenocarcinoma. Gastrointest Endosc..

[14] Kloth C., Fabricius D., Wendlik I., Schmidt S.A., Pfahler M., Lormes E., Beer M., Kratzer W., Schmidberger J. (2019). Diagnostic accuracy of MRI with MRCP and B-Mode-sonography with elastography of the pancreas in patients with cystic fibrosis: A point-to-point comparison. BMC Res. Notes.

[15] Itoh Y., Itoh A., Kawashima H., Ohno E., Nakamura Y., Hiramatsu T., Sugimoto H., Sumi H., Hayashi D., Kuwahara T. (2014). Quantitative analysis of diagnosing pancreatic fibrosis using EUS-elastography (comparison with surgical specimens). J. Gastroenterol..

[16] Kawada N., Tanaka S. (2016). Elastography for the pancreas: Current status and future perspective. World J. Gastroenterol..

[17] Pozzi R., Parzanese I., Baccarin A., Giunta M., Conti C.B., Cantù P., Casazza G., Tenca A., Rosa R., Gridavilla D. (2017). Point shear-wave elastography in chronic pancreatitis: A promising tool for staging disease severity. Pancreatology.

[18] Deng Y., Zhao B., Yang M., Li C., Zhang L. (2018). Association Between the Incidence of Pancreatic Fistula After Pancreaticoduodenectomy and the Degree of Pancreatic Fibrosis. J. Gastrointest. Surg..

[19] Sinha A., Singh V.K., Cruise M., Afghani E., Matsukuma K., Ali S., Andersen D.K., Makary M.A., Raman S.P., Fishman E.K. (2015). Abdominal CT predictors of fibrosis in patients with chronic pancreatitis undergoing surgery. Eur. Radiol..

[20] Goertz R.S., Schuderer J., Strobel D., Pfeifer L., Neurath M.F., Wildner D. (2016). Acoustic radiation force impulse shear wave elastography (ARFI) of acute and chronic pancreatitis and pancreatic tumor. Eur. J. Radiol..

[21] D’Onofrio M., Gallotti A., Salvia R., Capelli P., Mucelli R.P. (2011). Acoustic radiation force impulse (ARFI) ultrasound imaging of pancreatic cystic lesions. Eur. J. Radiol..

[22] D’Onofrio M., Gallotti A., Mucelli R.P. (2010). Pancreatic Mucinous Cystadenoma at Ultrasound Acoustic Radiation Force Impulse (ARFI) Imaging. Pancreas.

[23] D’Onofrio M., Gallotti A., Martone E., Mucelli R.P. (2009). Solid appearance of pancreatic serous cystadenoma diagnosed as cystic at ultrasound acoustic radiation force impulse imaging. JOP J. Pancreas.

[24] Kaya M., Değirmenci S., Göya C., Tuncel E.T., Uçmak F., Kaplan M.A. (2018). The importance of acoustic radiation force impulse (ARFI) elastography in the diagnosis and clinical course of acute pancreatitis. Turk. J. Gastroenterol..

[25] Yashima Y., Sasahira N., Isayama H., Kogure H., Ikeda H., Hirano K., Mizuno S., Yagioka H., Kawakubo K., Sasaki T. (2012). Acoustic radiation force impulse elastography for noninvasive assessment of chronic pancreatitis. J. Gastroenterol..

[26] Llamoza-Torres C.J., Fuentes-Pardo M., Álvarez-Higueras F.J., Alberca-De-Las-Parras F., Carballo-Álvarez F. (2016). Usefulness of percutaneous elastography by acoustic radiation force impulse for the non-invasive diagnosis of chronic pancreatitis. Rev. Española Enfermedades Digestivas.

[27] Mateen M.A., A Muheet K., Mohan R.J., Rao P.N., Majaz H.M.K., Rao G.V., Reddy D.N. (2012). Evaluation of ultrasound based acoustic radiation force impulse (ARFI) and eSie touch sonoelastography for diagnosis of inflammatory pancreatic diseases. JOP.

[28] Kuwahara T., Hirooka Y., Kawashima H., Ohno E., Ishikawa T., Yamamura T., Furukawa K., Funasaka K., Nakamura M., Miyahara R. (2018). Usefulness of shear wave elastography as a quantitative diagnosis of chronic pancreatitis. J. Gastroenterol. Hepatol..

[29] Pfahler M.H.C., Kratzer W., Leichsenring M., Graeter T., Schmidt S.A., Wendlik I., Lormes E., Schmidberger J., Fabricius D. (2018). Point shear wave elastography of the pancreas in patients with cystic fibrosis: A comparison with healthy controls. Abdom. Radiol..

[30] D’Onofrio M., Tremolada G., De Robertis R., Crosara S., Ciaravino V., Cardobi N., Marchegiani G., Pulvirenti A., Allegrini V., Salvia R. (2018). Prevent Pancreatic Fistula after Pancreatoduodenectomy: Possible Role of Ultrasound Elastography. Dig. Surg..

[31] Huang C.-T., Liang Y.-J. (2019). Comparison of Duodenal Mucosal Chromogranin-A Expression in Non-Alcoholic Fatty Pancreas Dyspeptic Patients with and without Endosonography-Diagnosed Early Chronic Pancreatitis: A Case Series Study. Case Rep. Gastroenterol..

[32] Dietrich C.F., Hocke M. (2019). Elastography of the Pancreas, Current View. Clin. Endosc..

[33] Catalano M.F., Sahai A., Levy M., Romagnuolo J., Wiersema M., Brugge W., Freeman M., Yamao K., Canto M., Hernandez L.V. (2009). EUS-based criteria for the diagnosis of chronic pancreatitis: The Rosemont classification. Gastrointest. Endosc..

[34] Trikudanathan G., Munigala S., Barlass U., Malli A., Han Y., Sekulic M., Bellin M., Chinnakotla S., Dunn T., Pruett T. (2017). Evaluation of Rosemont criteria for non-calcific chronic pancreatitis (NCCP) based on histopathology—A retrospective study. Pancreatol.

[35] Shimosegawa T., Kataoka K., Kamisawa T., Miyakawa H., Ohara H., Ito T., Naruse S., Sata N., Suda K., Hirota M. (2010). The revised Japanese clinical diagnostic criteria for chronic pancreatitis. J. Gastroenterol..

[36] Masamune A., Nabeshima T., Kikuta K., Hamada S., Nakano E., Kume K., Kanno A., Sato A., Tachibana Y., Inatomi O. (2019). Prospective study of early chronic pancreatitis diagnosed based on the Japanese diagnostic criteria. J. Gastroenterol..

[37] Hashimoto S., Futagami S., Yamawaki H., Kaneko K., Kodaka Y., Wakabayashi M., Sakasegawa N., Agawa S., Higuchi K., Akimoto T. (2017). Epigastric pain syndrome accompanying pancreatic enzyme abnormalities was overlapped with early chronic pancreatitis using endosonography. J. Clin. Biochem. Nutr..

[38] Wakabayashi M., Futagami S., Yamawaki H., Tatsuguchi A., Kaneko K., Agawa S., Higuchi K., Sakasegawa N., Murakami M., Akimoto T. (2018). Comparison of clinical symptoms, gastric motility and fat intake in the early chronic pancreatitis patients with anti-acid therapy-resistant functional dyspepsia patients. PLoS ONE.

[39] Yamawaki H., Futagami S., Kaneko K., Agawa S., Higuchi K., Murakami M., Wakabayashi M., Sakasegawa N., Kodaka Y., Ueki N. (2019). Camostat Mesilate, Pancrelipase, and Rabeprazole Combination Therapy Improves Epigastric Pain in Early Chronic Pancreatitis and Functional Dyspepsia with Pancreatic Enzyme Abnormalities. Digestion.

[40] Tirkes T., Yadav D., Conwell D.L., Territo P.R., Zhao X., Venkatesh S.K., Kolipaka A., Li L., Pisegna J.R., Pandol S.J. (2019). Magnetic resonance imaging as a non-invasive method for the assessment of pancreatic fibrosis (MINIMAP): A comprehensive study design from the consortium for the study of chronic pancreatitis, diabetes, and pancreatic cancer. Abdom. Radiol. (N.Y.).

[41] Bieliuniene E., Frøkjær J.B., Pockevicius A., Kemesiene J., Lukosevičius S., Basevicius A., Atstupenaite V., Barauskas G., Ignatavicius P., Gulbinas A. (2019). CT- and MRI-Based Assessment of Body Composition and Pancreatic Fibrosis Reveals High Incidence of Clinically Significant Metabolic Changes That Affect the Quality of Life and Treatment Outcomes of Patients with Chronic Pancreatitis and Pancreatic Cancer. Medicina.

[42] Park W.G., Li L., Appana S., Wei W., Stello K., Andersen D.K., Hughes S.J., Whitcomb D.C., Brand R.E., Yadav D. (2020). Unique circulating immune signatures for recurrent acute pancreatitis, chronic pancreatitis and pancreatic cancer: A pilot study of these conditions with and without diabetes. Pancreatology.

[43] Oh H.-C., Kwon C.-I., El Hajj I.I., Easler J.J., Watkins J., Fogel E.L., McHenry L., Sherman S., Zimmerman M.K., Lehman G.A. (2017). Low Serum Pancreatic Amylase and Lipase Values Are Simple and Useful Predictors to Diagnose Chronic Pancreatitis. Gut Liver.

[44] Kwon C.-I., Kim H.J., Korc P., Choi E.K., McNulty G.M., Easler J.J., El Hajj I.I., Watkins J., Fogel E.L., McHenry L. (2016). Can We Detect Chronic Pancreatitis With Low Serum Pancreatic Enzyme Levels?. Pancreas.

[45] Rossi R.E., Ciafardini C., Sciola V., Conte D., Massironi S. (2018). Chromogranin A in the Follow-up of Gastroenteropancreatic Neuroendocrine Neoplasms: Is It Really Game Over? A Systematic Review and Meta-analysis. Pancreas.

[46] Rogowski W., Wachuła E., Lewczuk A., Kolesińska-Ćwikła A., Iżycka-Świeszewska E., Sulżyc-Bielicka V., Ćwikła J.B. (2017). Baseline chromogranin A and its dynamics are prognostic markers in gastroenteropancreatic neuroendocrine tumors. Futur. Oncol..

[47] Malaguarnera M., Cristaldi E., Cammalleri L., Colonna V., Lipari H., Capici A., Cavallaro A.S., Beretta M., Alessandria I., Luca S. (2009). Elevated chromogranin A (CgA) serum levels in the patients with advanced pancreatic cancer. Arch. Gerontol. Geriatr..

[48] Lee S.H., Jo J.H., Kim Y.J., Lee H.S., Chung M.J., Park J.Y., Bang S., Park S.W., Song S.Y. (2019). Plasma Chromogranin A as a Prognostic Marker in Pancreatic Ductal Adenocarcinoma. Pancreas.

[49] Cory M., Moin A.S.M., Moran A., A Rizza R., Butler P.C., Dhawan S., E Butler A. (2018). An Increase in Chromogranin A-Positive, Hormone-Negative Endocrine Cells in Pancreas in Cystic Fibrosis. J. Endocr. Soc..

[50] Moin A.S.M., Cory M., Choi J., Ong A., Dhawan S., Dry S.M., Butler P.C., Rizza R.A., Butler A.E. (2018). Increased Chromogranin A–Positive Hormone-Negative Cells in Chronic Pancreatitis. J. Clin. Endocrinol. Metab..

